# Effects of phosphogypsum on enzyme activity and microbial community in acid soil

**DOI:** 10.1038/s41598-023-33191-2

**Published:** 2023-04-16

**Authors:** Changan Li, Yonggang Dong, Yun Yi, Juan Tian, Chao Xuan, Yan Wang, Yuanbo Wen, Jianxin Cao

**Affiliations:** 1 Key Laboratory of Guizhou Province for Green Chemical Industry and Clean Energy Technology, School of Chemistry and Chemical Engineering, Guizhou University, Guiyang, 550025 Guizhou China; 2 School of Chemistry and Chemical Engineering, Qiannan Normal University for Nationalities, Duyun, 558000 Guizhou China; 3 Engineering Research Center of Efficient Utilization for Industrial Waste, Guizhou University, Guiyang, 550025 Guizhou China; 4 Guizhou Research Institute of Chemical Industry, Guiyang, 550002 Guizhou China

**Keywords:** Ecology, Environmental sciences

## Abstract

Phosphogypsum (PG) is a solid waste produced from decomposition of phosphate rock in sulfuric acid. It can improve the physicochemical properties of soil. However, the application of PG will inevitably change the living environment of soil microorganisms and lead to the evolution of the soil microbial community. The effects of PG (0, 0.01%, 0.1%, 1%, 10% PG) on soil respiration, enzyme activity and microbial community were studied systematically by indoor incubation experiments. The results showed that the addition of 0.01% PG had little effect on the soil physicochemical properties and microflora. The soil respiration rate decreased with the increase of PG; The activities of catalase, urease and phosphatase were decreased and the activities of sucrase were increased by 10% PG treatment, while 0.01% or 0.1% PG treatment improve the urease activity; Soil microbial community response was significantly separated by amount of the PG amendment, and the application of 10% PG reduced the abundance, diversity and evenness of soil bacteria and fungi. Redundancy analysis (RDA) showed that soil bacterial composition was mainly driven by electrical conductivity (EC) and Ca^2+^, while fungal composition was mainly driven by F^−^ and NH_4_^+^. In addition, the application of PG increased the abundance of salt-tolerant microorganisms and accelerated the degradation of soil organic matter. Overall, These results can help to revisit the current management of PG applications as soil amendments.

## Introduction

Phosphogypsum (PG) is a solid waste produced by sulfuric acid decomposing phosphate rock to produce phosphoric acid. The main component is calcium sulfate and contains some harmful impurities such as soluble phosphorus, fluorine and heavy metals. Therefore, PG has a high environmental risk. At present, the world produces about 300 million tons of PG every year, including about 70 million tons in China^1,2^, but the recycling rate of PG is only about 30%, and a large amount of PG is still mainly treated by stacking or landfill^3^. Therefore, the research on PG utilization has always been a hot spot in scientific research^4^.

As a soil conditioner, PG is one of the ways of its resource utilization^5^. PG can not only be used to improve saline-alkali land^6^, but also acid soil^7^. Soil acidity affects agricultural development in extensive areas around the world. Low fertility, Al^3+^ toxicity and Ca^2+^ deficiency are considered as the key influence factors of acid soils. PG can supplement calcium, sulfur and phosphorus in acid soil. In addition, the exchangeable Al^3+^ can be replaced by the cation from PG; then the Al^3+^ combines with the anion from PG to form AlSO_4_^+^, AlF_2_^+^, and AlF_3_, thus decreasing Al^3+^ toxicity^7–9^. However, some elements (phosphorus, fluorine and heavy metals) from PG may cause environmental risks through accumulation in soil and crops, as these elements exceed the allowable environmental value. Therefore, attention should be paid to the impact on soil ecology when applying PG in the agricultural field.

At present, a large number of scholars have done a lot of research work in the application of PG as a soil conditioner, which mainly focuses on the application effects of soil physical and chemical properties and plant growth, but the research on the impact of PG on soil microbial ecology is not very sufficient. Soil microorganisms are an important part of the soil ecosystem. They directly participate in the decomposition of soil organic matter, humus synthesis, nutrient transformation and promote soil formation and development. Soil enzymes are important participants in the metabolic process of soil ecosystem. Soil biochemical reactions are closely related to enzyme catalysis. In addition, Soil microbial community is extremely sensitive to soil environment changing, which might result in a dramatic effect on ecosystem functions. Meanwhile, Microorganisms also play a vital role in soil formation and quality, promoting plant growth and as plant pathogens. Microbial communities are actively associated with various pollutants transformation and degradation, which play a crucial role in the remediation processes.

Guizhou in China is located in a subtropical monsoon humid climate zone, with an annual precipitation of 1060–1200 mm and more soil acidification. In addition, China's Guizhou has great pressure on the healthy development of Guizhou phosphorus chemical enterprises and the surrounding ecological environment due to discharges about 11 million tons of PG every year. Therefore, in order to solve the problems of PG stockpiling and soil acidification, we tried to use PG to improve acid soil, based on a lot of research work done by many scholars on the application of PG as an acid soil conditioner. However, in PG-applied soils, there is insufficient research on how microbial communities respond to the environmental changes and how dominant soil microbes affect the ecosystem function. Therefore, it is necessary to further evaluate the impact of PG on soil microbial ecology. The aim of this study is to: (1) evaluate the effect of PG on soil respiration and enzyme activity; (2) describe the structure and diversity of soil bacterial communities under different PG doses; (3) Explore the environmental factors that form soil bacterial communities. The research results not only provide guidance for the management of PG in this area, but also provide a reference for PG to improve acid soil.

## Materials and methods

### Collection of PG and soil samples

The gray PG powder was obtained from a factory in Guizhou, China. The main component of PG was dihydrate gypsum (CaSO_4_·2H_2_O). It presented acidity as it contained a small amount of free phosphoric acid, soluble fluoride and sulfate (Table S6). Furthermore, there is a little organic matter and heavy metals in PG (Tables S5, S6). The pH was 3.20. The raw PG was dried in the oven at 40 °C for 8 h to remove the free water, then ground and sieved through 2 mm mesh, and sealed for storage. The soil was collected from the acid soil in Guiyang, Guizhou, China. Following the five-point method, the sampling soil was sourced from the top 0-20 cm of the tillage layer. The acid soil sample was air-dried, weeds-removed, ground through a 2 mm sieve, thoroughly blended and sealed for storage.

### Experimental design

The results of previous studies showed that the amount of PG in the range of 0 ~ 10% was beneficial to improve the physical and chemical properties of acid soil^10–13^. Hence, five PG-treated soil samples, Control (no PG), P1 (0.01% PG), P2 (0.1% PG), P3 (1% PG) and P4 (10% PG) were designed in the experiment. 200 g dry weight of soil and PG mixture was prepared in a 500 mL box for each treatment. It was then adjusted with distilled water to 40% moisture content and covered with a lid. The lid was pierced with 4 small holes to ensure air circulation in the box. Every treatment group had 3 repetitions. All the sample boxes were placed in an artificial climate incubator for laboratory cultivation. The incubator was maintained at 25 °C and a humidity of 80%. During the incubation, water was added to the boxes every 10 days to keep the soil moisture content constant. After 90 days of incubation, it was taken out from the artificial climate incubator and divided into three parts. The first part was naturally air-dried to determine soil physical and chemical properties; the second part was stored in a refrigerator at 4 °C for the determination of soil enzyme activity; The third part was stored in a − 30 °C refrigerator for high-throughput sequencing analysis of soil bacteria and fungi.

### Determination of soil physico-chemical properties, soil respiration, soil enzyme activity

Determination of soil physico-chemical properties: The soil active acidity value (pH (H_2_O)) was measured by pH meter (soil: water = 1:2.5); the soil was treated with 1 mol/L KCl (1:2.5 soil: water), and then the soil potential acidity (pH (KCl)) was measured by pH meter. Soil electrical conductivity (EC) was measured by an electrical conductivity meter (1:5 soil: water). Soil organic matter (SOM) was determined by potassium dichromate titration (NY/T 1121.6–2006). After the soil samples were discomposed by perchloric acid and sulfuric acid, the total phosphorus (TP) in soil was determined by molybdenum antimony anti-spectrophotometry. Available phosphorus (AP) was determined by hydrochloric acid ammonium fluoride molybdenum antimony anti-spectrophotometry (NY/T 1121.7-2014). Ammonium nitrogen (NH_4_^+^-N) was determined by KCl extraction indophenol blue colorimetry. Nitrate nitrogen (NO_3_^–^N) was determined by dual-wavelength UV colorimetry. Ca^2+^ was determined by atomic absorption spectrometry. Sulfate (SO_4_^2−^) was determined by Barium Sulfate Turbidimetry. Water-soluble fluorine ion (F^−^) was determined by the ion-selective electrode method. Each sample was set with 3 repetitions.

Determination of soil respiration: When the soil was treated with PG for 90 days, 50.00 g of fresh soil was weighed into a 500 mL jar with evenly spread on the bottom. 10 mL of 1 mol/L NaOH solution was placed into a 25 mL small beaker in the jar to capture the carbon dioxide (CO_2_) generated by soil respiration, and the jar was sealed to incubate at 28 °C for 24 h. Then the beaker containing NaOH solution was added 10 mL 1.0 mol/L BaCl_2_ solution and 2 drops phenolphthalein indicator to titrate with 0.500 mol/l HCl standard solution to the end point (pink turns colorless). The carbon dioxide content was calculated according to the amount of HCl. Each sample was set with 3 repetitions, and a blank control was used at the same time.

Determination of soil enzyme activity: Soil urease activity was measured by phenol sodium colorimetry, expressed in mg of NH_3_-N in 1 g soil after 24 h^14^. Invertase activity was measured by 3,5-dinitrosalicylic acid colorimetry and expressed in mg of glucose produced by 1 g dry soil in 24 h^15^. Catalase was expressed by the volume (mL) of 0.1 mol/L KMnO_4_ consumed by 1 g of dry soil in 1 h^16^. Phosphatase activity was determined by the mass (μg) of phenol released from 1 g soil after 24 h using sodium diphenyl phosphate colorimetry^17^.

### Extraction and sequencing of total DNA from soil and analysis of microbial community

Microbial DNA from soil samples was extracted using FastDNA spin kit for soil (MP biomedicals, Santa Ana, CA). The quantity and quality of extracted DNA were measured by ultra micro spectrophotometer (NanoDrop 2000, Thermo Scientific, USA) and a gel electrophoresis instrument (DYY-6C, Beijing Liuyi, China). PCR amplification was performed on the V3–V4 region of the bacterial 16S rRNA gene with the primer pair 338F (5'-ACTCCTACGGGAGGCAGCA-3') and 806R (5'-GGACTACHVGGGTWTCTAAT-3'). The fungal ITS-rRNA gene was amplified by PCR. The primer pair was ITS5F (5'-GGAAGTAAAAGTCGTAACAAGG′-3') and ITS1R (5'-GCTGCGTTCTTCATCGATGC-3').

The 25 μL reaction system for the amplification process contained 5 μL of 5 × reaction buffer, 5 μL of 5 × GC buffer, 2 μL (2.5 mM) dNTPs, 1 μL (10 μM) of forward primer, 1 μL (10 μM) of reverse primer, 2 μL of DNA template, 8.75 μL of distilled water, and 0.25 μL of Q5 DNA polymerase.

The PCR amplification program included the initial denaturation at 98 °C for 2 min, denaturation at 98 °C for 15 s, annealing at 55 °C for 30 s and extension at 72 °C for 30 s. The cycle was repeated 25–30 times followed by a final extension at 72 °C for 5 min. The resulting PCR product was stored at 10 °C.

The PCR amplicon was purified by Agencourt AMPure Beads (Beckman Coulter, Indianapolis, IN, USA) reagent. The quantity of PCR amplicon was determined by PicoGreen dsDNA Assay Kit (Invitrogen, Carlsbad, CA, USA). The same amount of mixed amplicons was sequenced in Shanghai Personal Biotechnology Co., Ltd. The 2 × 300 paired-end sequencing was performed on the Illumina Novaseq-PE250 platform using the MiSeq Reagent Kit v3.

### Data analysis

Microsoft Excel 2010 was used for statistics and calculation, SPSS version 26.0 was used for significance and correlation analysis, and Origin 2021b software was used for graphing. One-way ANOVA and Duncan multiple comparisons were used to test the difference significance between different treatment groups (P < 0.05). Pearson correlation coefficient was used for correlation analysis. The free online platform GenesCloud (https://www.genescloud.cn) was used to analyse the high throughput sequencing data and visualise the OTUs of each sample.

## Results

### Effect of PG on soil physical and chemical properties

The application of PG significantly changed the physical and chemical properties of acidic soil (Table 1). Almost all of the soil properties changed significantly at given PG applications. For example, for P2 (PG = 0.1%), the content of AP, TP, NH_4_^+^-N, NO_3_^–^N, Ca^2+^, SO_4_^2−^ and EC increase significantly. For P3 (PG = 1%) and P4 (PG = 10%), in addition to the above soil components and EC, pH (KCl) and water-soluble F^−^ also significantly increased; meanwhile, pH (H_2_O) and soil organic matter (SOM) content were significantly reduced.

**Table 1:** Soil properties under different treatments.

Sample	Control	P1	P2	P3	P4
pH (H_2_O)	4.62 ± 0.02a	4.60 ± 0.01a	4.50 ± 0.03b	4.44 ± 0.02c	4.63 ± 0.01a
pH (KCl)	3.85 ± 0.01c	3.83 ± 0.03c	3.85 ± 0.01c	3.91 ± 0.02b	4.06 ± 0.01a
EC (μS/cm)	103.33 ± 0.64d	108.13 ± 5.90d	201.00 ± 1.73c	866.67 ± 4.04b	1528.00 ± 6.00a
AP (mg/kg)	26.59 ± 0.91d	27.03 ± 0.44d	29.36 ± 0.04c	50.28 ± 0.88b	349.41 ± 2.38a
TP (mg/kg)	525.62 ± 6.55d	524.04 ± 9.03d	539.41 ± 5.61c	589.69 ± 5.61b	888.83 ± 5.85a
NH_4_^+^-N (mg/kg)	45.39 ± 2.68c	42.60 ± 0.86c	45.34 ± 5.06c	75.02 ± 0.86a	52.70 ± 2.74b
NO_3_^–^N (mg/kg)	45.01 ± 0.26c	43.89 ± 0.32c	47.85 ± 1.97b	52.29 ± 0.72a	47.46 ± 1.91b
SOM (g/kg)	29.29 ± 0.60a	29.20 ± 0.64a	28.41 ± 0.40ab	27.93 ± 0.40b	26.27 ± 0.34c
Ca^2+^ (mg/kg)	20.71 ± 1.52c	19.31 ± 0.03c	22.76 ± 3.52c	113.19 ± 0.71b	140.90 ± 1.36a
SO_4_^2−^ (g/kg)	0.80 ± 0.22c	0.78 ± 0.12c	1.23 ± 0.07c	6.31 ± 0.60b	24.55 ± 0.38a
F^−^ (mg/kg)	19.92 ± 0.58c	19.92 ± 0.58c	13.22 ± 0.12c	70.60 ± 5.13b	415.23 ± 18.63a

Different lowercase letters stand for significant differences at the level of P < 0.05. Control: no PG was applied; P1: 0.01% PG; P2: 0.1% PG; P3: 1% PG; P4: 10% PG.

### Effect of PG application on soil respiration

**Figure 1:**
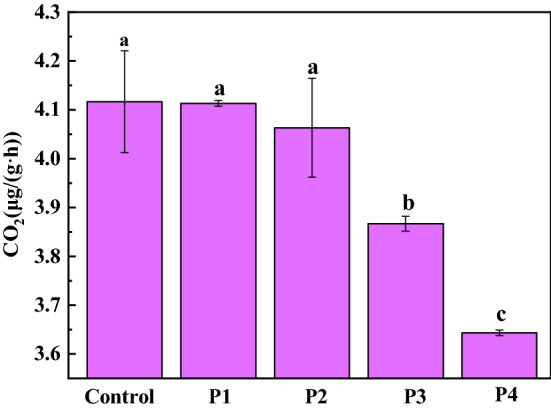
Effect of PG on soil respiration. Different lowercase letters represent significant differences at the level of P < 0.05.

The doses of PG treatment affected the soil respiration rate (Fig. 1). After PG was applied, there was no significant difference in soil respiration rates between the control, P1 and P2. But the soil respiration rates of P3 and P4 decreased by 6.07% and 11.65% respectively, compared to the control.

### Effect of PG application on soil enzyme activity

**Figure 2:**
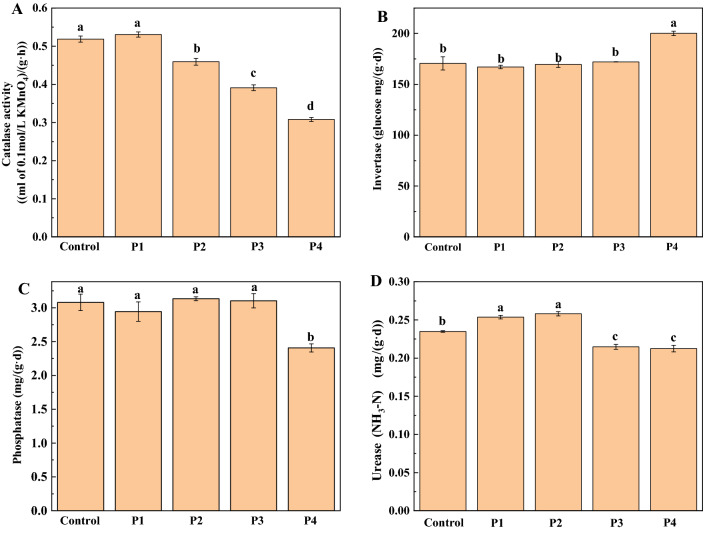
Effect of PG on soil enzyme activity. Catalase (**A**), Invertase (**B**), Phosphatase (**C**) and Urease (**D**). Different lowercase letters represent significant differences at the level of P < 0.05.

The doses of PG treatment affected soil enzyme activity (Fig. 2). Catalase activity decreased significantly with the increase of PG application (except P1) (Fig. 2A). The application of 0.01% or 0.1% PG increased significantly urease activity (P1 and P2), and 1% or 10% PG inhibited significantly urease activity (P3 and P4) (Fig. 2D). Compared with the control, there was no significant difference in soil phosphatase and invertase (P1, P2 and P3) when 0.01% ~ 1% PG was applied. When 10% PG was applied, soil phosphatase activity decreased prominently 21.75%, and soil invertase activity increased by 17.26% (Fig. 2B,C).

**Figure 3:**
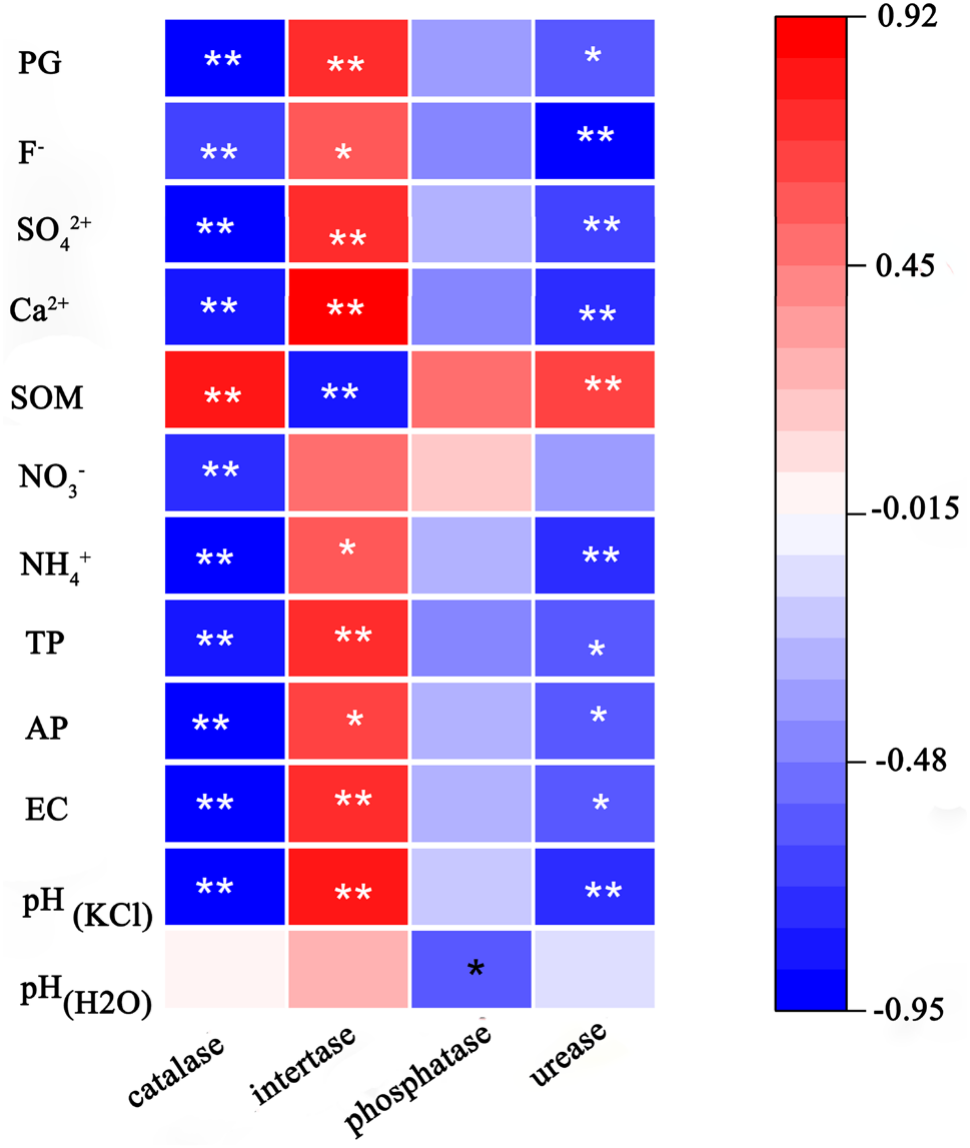
Correlation between soil enzyme activity and soil characteristics. * P < 0.05; ** P < 0.01.

The correlation analysis between soil enzyme activity and soil properties is shown in Fig. 3. Catalase had a positive correlation with SOM (P < 0.01), a negative correlation with PG, F^−^, SO_4_^2+^, Ca^2+^, NO_3_^–^N, NH_4_^+^-N, TP, AP, EC and pH (KCl) (P < 0.01), and no significant correlation with pH (H_2_O) (P > 0.05). Invertase was positively correlated with F^−^, NH_4_^+^-N and AP (P < 0.05), positively correlated with PG, SO_4_^2+^, Ca^2+^, TP, EC and pH (KCl) (P < 0.01), negatively correlated with SOM (P < 0.01), but not significantly correlated with NO_3_^–^N and pH (H_2_O) (P > 0.05). Phosphatase was merely negatively correlated with pH (H_2_O) (P < 0.05). Urease was positively correlated with SOM (P < 0.01), negatively correlated with PG, TP, AP and EC (P < 0.05), negatively correlated with F^−^, SO_4_^2+^, Ca^2+^, NH_4_^+^-N and pH (KCl) (P < 0.01), and not significantly correlated with NO^3–^N and pH (H_2_O) (P > 0.05).

### Effects of PG on soil bacterial and fungal communities

#### Community composition of soil bacteria and fungi

**Figure 4:**
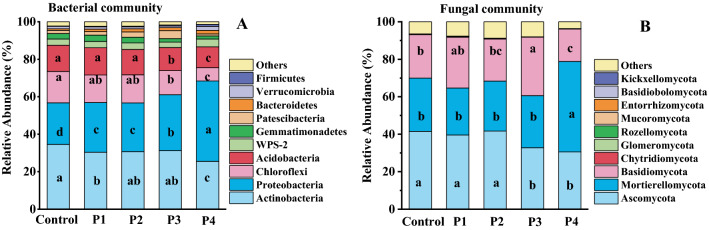
Effect of PG on the relative abundance of bacterial (**A**) and fungal (**B**) communities at the phylum level. Different lowercase letters represent significant differences at the level of P < 0.05.

With the increase of PG content, although the compositions of dominant bacterial taxa in soil at the phyla level are similar, the relative abundance of some phyla is different (Fig. 4A and Table S1). The dominant bacterial phyla (> 5% relative abundance) in the control and PG-treated soil are *Actinobacteria*, *Proteobacteria*, *Chloroflexi* and *Acidobacteria*, with relative abundances of 25.6–34.6%, 22.2–42.9%, 7.1–16.8% and 11.1–14.5% respectively (Fig. 4A and Table S1). Among the four dominant bacterial phyla, P4 (10% PG) reduced the abundance of *Actinobacteria*, *Acidobacteria* and *Chloroflexi* by up to 26.01%, 57.74% and 20.72% respectively and also increased the relative abundance of *Proteobacteria* by 93.24% compared to the control group.

With the increase of PG content, although the compositions of dominant fungal phyla in soil are similar, the relative abundance of some fungal phyla is altered, and the P4 treatment has the greatest impact (Fig. 4B and Table S2). The dominant fungal phyla (> 5% relative abundance) in PG-treated soil are *Ascomycota* (30.54–41.53%), *Mortierellomycota* (25.05–48.36%) and *Basidiomycota* (17.32–27.08%), altogether making a total relative abundance of 89.07–96.63% (Fig. 4B and Table S2). Compared with the control, 10% PG treatment significantly reduced the relative abundance of *Ascomycota* and *Basidiomycota* communities, but it significantly enhanced the relative abundance of *Mortierellomycota*.

#### Soil microbial alpha diversity analysis

Microbial alpha diversity refers to the abundance, diversity and evenness of microbial species in a specific area. Different alpha diversity indexes reflect different information of microorganisms. In this experiment, Chao1, Shannon and Pielou's indexes were selected to reflect the abundance, diversity and evenness of the microbial community.

The experiment showed that the doses of PG treatment affected the abundance, diversity or evenness of soil bacterial and fungal communities (Table 2). When 1% PG was applied (P3), the soil bacterial abundance, diversity and evenness decreased significantly by 11.57%, 3.25% and 2.33%, and the soil fungal diversity decreased significantly by 9.97%. When 10% PG was applied (P4), the soil bacterial abundance, diversity and evenness decreased significantly by 30.07%, 9.16% and 4.65%, and the soil fungal abundance, diversity, and evenness decreased significantly by 29.71%, 19.77%, and 14.93% respectively.

**Table 2:** Effect of PG on the alpha diversity of soil bacteria and fungal communities.

Treatments	Bacterial community			Fungal community		
	Chao1	Shannon	Pielou’s	Chao1	Shannon	Pielou’s
Control	4144.95 ± 162.40ab	10.15 ± 0.06a	0.86 ± 0.00a	649.00 ± 27.71a	6.22 ± 0.22a	0.67 ± 0.02ab
P1	4394.51 ± 224.97a	10.23 ± 0.05a	0.86 ± 0.00a	611.62 ± 109.17a	6.26 ± 0.08a	0.68 ± 0.01a
P2	4369.19 ± 548.36a	10.15 ± 0.18a	0.86 ± 0.01a	583.67 ± 45.84a	6.11 ± 0.49ab	0.67 ± 0.05ab
P3	3665.39 ± 65.20b	9.82 ± 0.11b	0.84 ± 0.00b	533.93 ± 55.43ab	5.60 ± 0.29b	0.62 ± 0.02b
P4	2898.57 ± 138.99c	9.22 ± 0.07b	0.82 ± 0.01b	456.16 ± 35.11b	4.99 ± 0.16c	0.57 ± 0.01c

Different lowercase letters represent significant differences at the level of P < 0.05.

#### Beta diversity analysis of soil microorganisms

**Figure 5:**
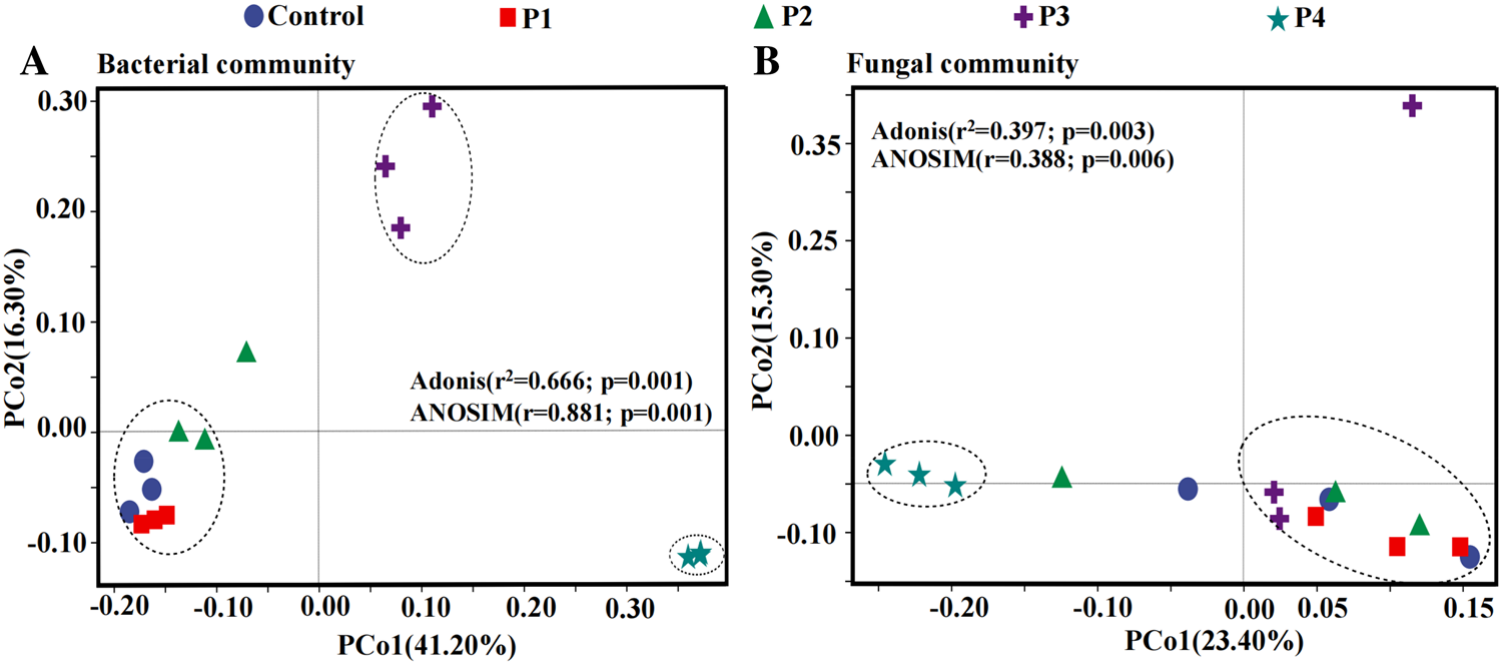
PCoA analysis of soil bacteria (**A**) and fungi (**B**) under different application rates of PG.

Principal coordinate analysis (PCoA) based on Bray Curtis distance showed that there were significant differences between treatment groups in both soil bacterial (Fig. 5A) and fungal (Fig. 5B) communities.

In Fig. 5A, ANOSIM (r = 0.881; P = 0.001) and Adonis (R^2^ = 0.666; P = 0.001) confirmed that the differences of bacterial communities between groups were larger than within-taxa. The distance between sample points showed that the bacterial communities of the control, P1 and P2 were clustered, while those of P3 and P4 were clustered respectively, suggesting 1–10% PG affected the bacterial community structure more significantly than the lower amount of PG (0.01–0.1%). In order to determine specific bacterial taxa enriched within the different treatments, LEfSe from the phylum to genus level was performed (Fig. S1). For example, the phyla *Actinobacteria* and *Chloroflexi* in Control; the phyla *Elusimicrobia* and *Acidobacteria* in P1 treatment; the phylum of *Patescibacteria* in P3 treatment; the phyla of *Bacteroidetes*, *Firmicutes*, *Proteobacteria* and *Verrucomicrobia* in P4 treatment were all significantly enriched. The above information only includes microbial community analysis at the phylum level; Fig. S1 shows the bacterial communities enriched significantly at different taxonomic levels for each treatment.

In Fig. 5B, ANOSIM (r = 0.388; P = 0.006) and Adonis (R^2^ = 0.397; P = 0.003) indicated that the differences of fungal communities were also higher between groups than within-group. Fungal communities of control, P1, P2 and P3 treatment taxa were clustered, while the cluster of P4 was isolated. This showed that the 10% PG led to a acute change in the fungal community structure. According to LEfSe analysis (Fig. S2), the family *Geminibasidiaceae* in Control; the family *Agaricaceae* in P1 treatment; the families *Diaporthaceae* and *Atheliaceae* in P2 treatment; and the family of *Sclerodermataceae* in P3 treatment was enriched. No fungal taxa were significantly enriched at the family level in P4 treatment. The fungal community data onto each treatment of any clade is shown in Fig. S2.

In all, PG doses significantly changed the community composition of bacteria and fungi in acidic soil. The differences were more pronounced when the highest PG dose was used. In addition, the bacterial community was more sensitive to the application of PG than the fungal community.

#### Relationship between soil microbial community structure and environmental factors

**Figure 6:**
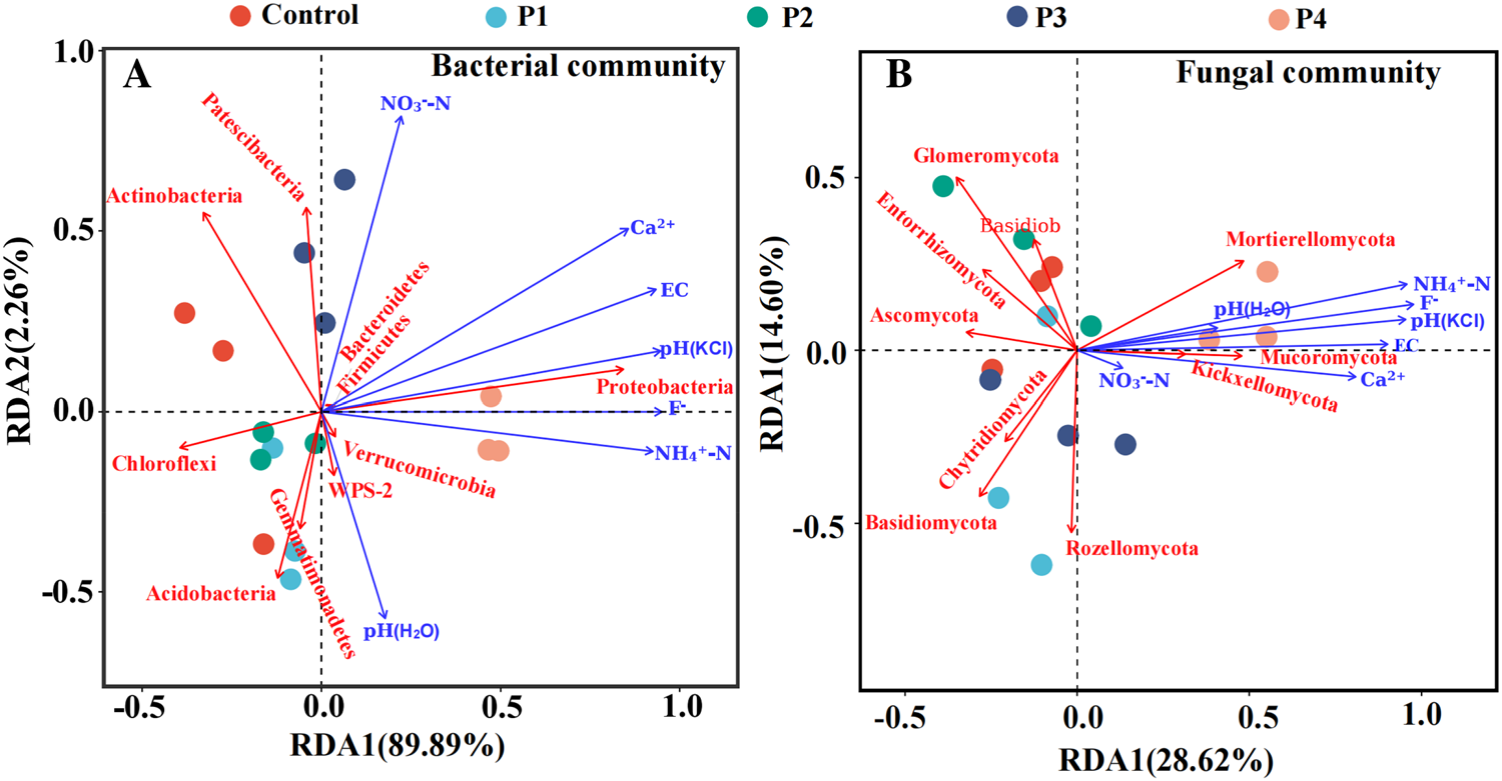
RDA (redundancy analysis) of environmental factors and bacterial (**A**) and fungal (**B**) compositions.

The RDA results showed that (Fig. 6A and Table S3), the first and second ranking axes could explain 89.89% and 2.26% of the variation respectively in the bacterial composition. The soil bacterial composition was synergistically influenced by soil EC, Ca^2+^, pH (KCl), F^−^, NH^4+^-N, among which EC and Ca^2+^ were the main driving factors. Figure 6B and Table S4 showed that the first and second ranking axes explain 28.62% and 14.6% of the variation for the fungal composition. Moreover, for the soil fungal composition, the driven factors were F-, NH^4+^-N, pH (KCl), Ca^2+^ and EC, in which F^−^ and NH^4+^-N were the dominant factors.

## Discussion

### Effect of PG application on soil physical and chemical properties

PG has a high environmental risk because it owns some harmful impurities such as soluble phosphorus, fluorine and heavy metals. Table S5 shows that the contents of cadmium, arsenic, lead and chromium in PG are lower than those in Control, while the content of mercury is higher than that in Control, but lower than the risk screening value of China (pH ≤ 5.5, Hg concentration is 1.3 mg/kg). Therefore, the heavy metals in PG will not pose a threat to the soil environment. These findings agree with Pérez-López^18^, who reported that PG did not contain large amount of heavy metals and that addition of PG did not lead to soil contamination. Kassir et al.^19^ monitored the effect of PG application on heavy metals in Mediterranean red soil and showing that the exchangeable and acid soluble contents of heavy metals in PG applied soil were higher than those in PG-untreated soil. Our study found that the application of 0.1% PG reduced the content of water-soluble fluorine, and the application of 1% or 10% PG significantly increased the content of water-soluble fluorine, indicating that the appropriate application of PG will not lead to soil fluorine pollution. Cui et al.^20^ also reported that the F^−^ concentration on the leachate of PG-treated soils was lower than that in blank treatment. However, one possible explanation for the increase of water-soluble fluorine content could be that the PG carried F^−^ into soil (Table S6). Therefore, PG should be applied to the soil after harmless treatment.

This experiment found that the application of PG can improve the effective nutrients of acidic soil, such as AP, NH_4_^+^-N and NO_3_^–^N. Crusciol et al. also found that PG can boost the available NO_3_^–^N in 0–5 cm tropical no-tillage soil layer^7^. Kinjo & Pratt found that the content of NO_3_^−^ in soil solution increased linearly with the increase of SO_4_^2−^ due to the competitive effect of soil between SO_4_^2−^ and NO_3_^−^ adsorption^21^. Such a competition effect can explain the positive effect of PG on NO_3_^−^. It was found that PG could not improve the soil active acidity (pH (H_2_O)), but could promote the soil potential acidity (pH (KCl)). Previous studies also reported that PG was not an effective material to improve soil acidity, but Al^3+^ could react with SO_4_^2−^, F^−^ and PO_4_^3−^ in soil solution, resulting in decreased Al^3+^ in soil solution and the increase of soil potential acidity^22^.

### Effect of PG on soil respiration

The experimental data showed that the application of PG can inhibit soil respiration, which was equivalent to inhibiting CO_2_ emission. Such a result was consistent with the results of Wu^23^, which showed that PG treatment could inhibit the emission of greenhouse gas in wheat soil and reduce the emission of soil CO_2_ by 2.5%-6.6%. The research on using PG as a calcium source to store CO_2_ showed that PG could increase the effective calcium in the system^24^, and the effective calcium could fix the CO_2_ produced by the organic matter decomposition and hence reduce CO_2_ emission^25^.

### Effect of PG on soil enzyme activity

Soil enzyme activity is affected by soil physical and chemical properties, microorganisms, substrates and other factors^26^. Although the effects of PG on the activity of some soil enzymes (invertase, amylase and cellulase) have been studied^13^, the effects of PG on catalase, invertase, phosphatase and urease are not clear. This study found that the low addition of phosphogypsum (≤ 0.1% PG) improved soil urease activity, whereas excess PG would inhibit the activity of soil catalase, phosphatase and urease (except invertase). Sengupta and Dhal found that a mixture of 150 mg acid soil and 5.25 g PG reduced the soil microbial catabolic activity, but the soil microbial catabolic activity gradually recovered over time^11^. Therefore, future study should examine the dynamic changes of soil enzyme activity. Soil urease activity first increased and then decreased, consistent with the changing trend of bacterial abundance and diversity (Table 2), indicating that the soil bacterial community may affect the soil urease. The correlation analysis between soil enzyme activity and soil properties found that phosphatase was significantly positively correlated with pH (H_2_O), indicating that soil pH was the main factor affecting phosphatase activity. Unexpectedly, acid phosphatase activity was not significantly correlated with TP and AP, which might be mainly caused by the increase of TP and AP with the phosphate brought in by PG. Catalase, invertase and urease were significantly correlated with pH (KCl), SOM, Ca^2+^, SO_4_^2−^, F^−^. Previous studies have shown that the interaction between SO_4_^2−^ / F^−^ and Al^3+^ in soil affects soil acidity (pH (H_2_O) and pH (KCl))^27^, and Ca^2+^ had a strong impact on the storage and stability of soil organic matter (SOM)^28^. Therefore, it is speculated that Ca^2+^, SO_4_^2−^ and F^−^ ions may be the main influencing factors of soil enzyme activity. The reason for the decrease of soil enzyme activity may be that Ca^2+^, SO_4_^2−^ and F^−^ ions restrain the growth of microorganisms and hence reduce their ability to secrete enzymes, resulting in the impeded activity of soil enzymes. When microorganisms are inhibited, the sudden increase of invertase activity may be due to the increase of a specific sucrose decomposing microorganism.

### Effect of PG on soil microbial community

The dominant bacterial phyla in PG-treated soil are *Actinobacteria*, *Proteobacteria*, *Chloroflexi* and *Acidobacteria*. These results agree with Guo^29^, who reported that the dominant bacteria were *Proteobacteria*, *Chloroflexi*, *Actinobacteria* and *Actinobacteria* after application of CaCO_3_ (0, 2.25, 4.5, 7.5 t/hm^2^) in acid soils in southern China. It was reported that *Proteobacteria*, *Actinobacteria*, *Firmicutes* and *Bacteroidetes* were the dominant bacteria in PG, and these strains generally had good adaptability to extreme environments^30,31^. In the present study, the changes of bacterial community composition can be considered as adaptations to the altered soil environment, as application of PG changed soil physicochemical properties (Table 1). In addition, the relative abundances of *Bacteroidetes*, *Firmicutes*, *Proteobacteria* and *Verrucomicrobia* phyla were increased under 10% PG treatment (Fig. 4A) due to better salt tolerance of these strains^32^, which may help to improve the salt tolerance of plants^33^. The dominant fungal phylum in PG-treated soil is *Ascomycota*, *Mortierellomycota* and *Basidiomycota*. The bacterial community exhibited more significant changes than the fungal community (Figs. S1, S2). This is because bacterial communities are more responsive than fungal communities^34^.

The doses of PG treatment in acid soil can significantly affect soil microbial structure. The results of study showed that the abundance, diversity and evenness of soil bacteria and fungi decreased significantly with the increase of PG (Table 2). Redundancy analysis (RDA) was used to explore the relationship between bacterial and fungal communities and the environment. It was found that the soil EC and Ca^2+^ were the main driving factors for the evolution of bacterial communities, and F^−^ and NH_4_^+^-N were the primary driving factors for the fungal communities. Therefore, with increased PG, the microbial alpha diversity decreased due to the increase of soil EC, Ca^2+^ and F^−^. The impaired alpha diversity further resulted in the soil microbial community being subjected to salt stress, calcium stress and fluorine toxicity. Corwin and Yemoto reported a significant correlation between soil EC and salinity: the higher the EC, the higher the salinity^35^. Hence, the change in the microbial communities after the PG treatments may result from the salt stress (EC). In addition, studies have shown that a large amount of calcium entering the cells may cause cell apoptosis, necrosis or autophagy, leading to severe cell damage even death^36^. As an important second messenger, overloaded calcium ions in cells over-activate a variety of enzyme systems, destroy cell membranes and produce a large number of free radicals such as reactive oxygen species and reactive nitrogen, attack the integrity of cell mitochondria and genome, cause DNA damage and induce apoptosis^37^. Cristina et al.^38^ found that calcium ions inhibited microbial activity by destroying intercellular communication. Excessive use of PG resulted in the high concentration of water-soluble calcium ions in soil solution, which may have a cytotoxic effect and affect soil microbial diversity and abundance. Because the fluorine content of PG is higher than that of soil, F- content of the soil increases with the increase of PG application. However, a fluorine-containing environment is not conducive to the growth and reproduction of microorganisms. A low concentration of fluorine can inhibit microbial metabolism and growth, and high concentration can kill microorganisms^39^. Therefore, the diversity and abundance of bacteria and fungi in soil decreased.

### Effects of PG on soil organic matter

The composition of microbial communities plays a fundamental role on soil organic matter decomposition. *Proteobacteria* is considered crucial for the decomposition of lignocellulose and organic matter and *Firmicutes* play important roles in the decomposition of cellulose, hemicelluloses and lignin lignocellulose^40^. The decrease in soil organic matter contents may be due to the increased relative abundance of *Firmicutes* and *Proteobacteria* promoting the decomposition of soil organic matter. At the same time, the increase in invertase activity may be due to the secretion of more invertase by these dominant strains since more dominant strains were changed in the P4 treatment. Shifts in microbial communities and enzymatic activity may alter soil organic matter turnover and accumulation. In the future, it is important to conduct a more comprehensive evaluation of how phosphogypsum affects microorganisms and enzymes that participate in the carbon cycle, such as amylase, β-xylosidase, β-glucosidase, cellulase, and laccase. The experiment found that soil organic matter decreased, and invertase activity increased, indicating that the application of phosphogypsum may cause soil organic matter loss.

## Conclusion

This paper studied the effects of different PG application rates on soil respiration, soil enzyme activity and soil community, revealing the mechanism of PG on soil enzyme activity and microorganism. The soil respiration rate decreased with the increase of PG. 1% PG treatment had little effect on the soil physicochemical properties, and less than 20% on soil microbial indicators and enzyme activity. After 10% PG treatment, water-soluble fluoride increased 19.84 times, the activities of catalase, urease, and phosphatase decreased while invertase increased, but the abundance, diversity and evenness of soil bacteria and fungi reduced significantly. The dominant bacterial phyla are *Actinobacteria*, *Proteobacteria*, *Chloroflexi* and *Acidobacteria*, and the dominant fungal phyla are *Ascomycota*, Mortierellomycota and *Basidiomycota*. Redundancy analysis (RDA) showed that soil bacterial composition was mainly driven by electrical conductivity (EC) and Ca^2+^, while fungal composition was mainly driven by F^−^ and NH_4_^+^. These results can help to revisit the current management of PG applications as soil amendments, suggesting that appropriate application of PG can improve soil properties, but the negative effects of PG above a certain threshold level should be considered. Therefore, we suggest that the dose of PG to improve acid soil is less than 1%.

## Supplementary Information

Supplementary Information.

## Data Availability

The datasets used or analysed during the current study are available from the corresponding author on reasonable request.
