# Supplementary material for: Effects of phosphogypsum on enzyme activity and microbial community in acid soil
Source: Sci Rep. 2023 Apr 16;13:6189. doi: 10.1038/s41598-023-33191-2 (PMC10106453; doi:10.1038/s41598-023-33191-2)
Supplement: Supplementary file 1 — Supplementary Information. [file 41598_2023_33191_MOESM1_ESM.docx]

Supplementary Material

# Supplementary Figures and Tables

## Supplementary Figures


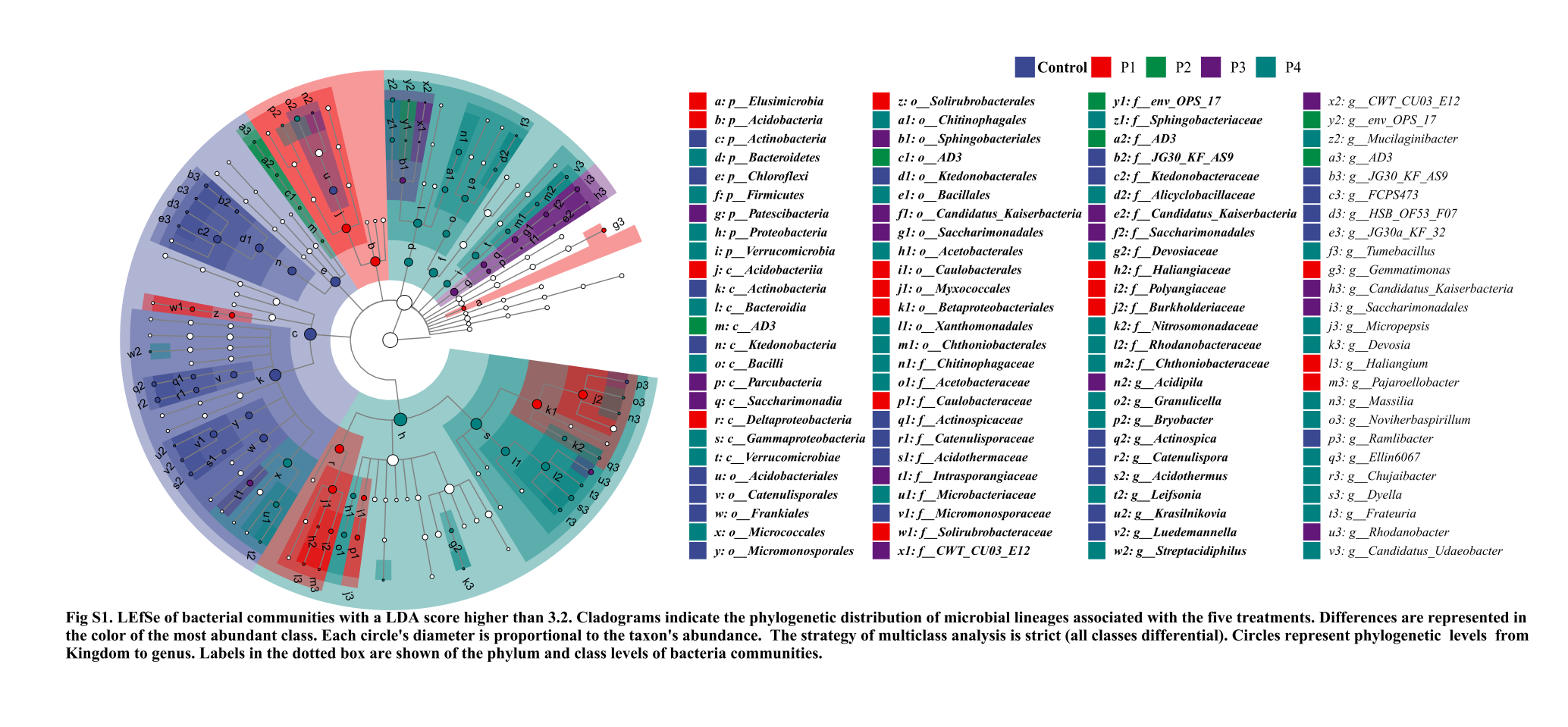


**Supplementary Figure 1.** LEfSe of bacterial communities with a LDA score higher than 3.2. Cladograms indicate the phylogenetic distribution of microbial lineages associated with the five treatments. Differences are represented in the color of the most abundant class. Each circle's diameter is proportional to the taxon's abundance. The strategy of multiclass analysis is strict (all classes differential). Circles represent phylogenetic levels from kingdom to genus. Labels in the dotted box are shown of the phylum and class levels of bacteria communities.


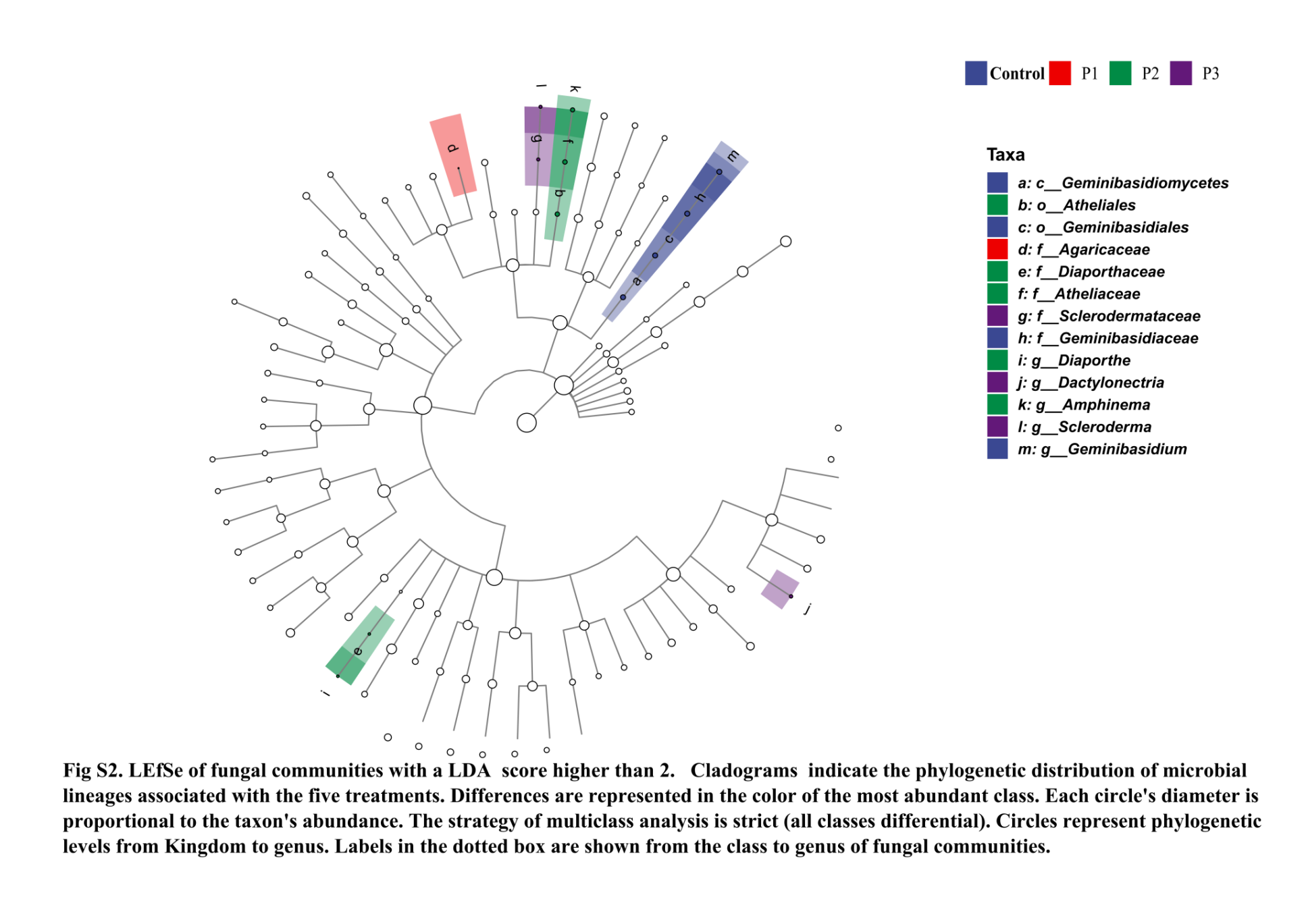


**Supplementary Figure 2.** LEfSe of fungal communities with a LDA score higher than 2. Cladograms indicate the phylogenetic distribution of microbial lineages associated with the five treatments. Differences are represented in the color of the most abundant class. Each circle's diameter is proportional to the taxon's abundance. The strategy of multiclass analysis is strict (all classes differential). Circles represent phylogenetic levels from Kingdom to genus. Labels in the dotted box are shown from the class to genus of fungal communities.

## Supplementary Tables

**Supplementary Table 1** Relative abundance of bacterial community in PG treated soil at the phylum level.

| Phyla | Control | P1 | P2 | P3 | P4 |
| --- | --- | --- | --- | --- | --- |
| *Actinobacteria* | 34.58 | 30.39 | 30.71 | 31.22 | 25.55 |
| *Proteobacteria* | 22.18 | 26.51 | 25.98 | 29.89 | 42.86 |
| *Chloroflexi* | 16.76 | 14.72 | 15.08 | 12.92 | 7.13 |
| *Acidobacteria* | 13.97 | 14.55 | 13.47 | 12.23 | 11.13 |
| *WPS-2* | 3.36 | 3.39 | 3.47 | 2.92 | 4.18 |
| *Gemmatimonadetes* | 2.96 | 3.32 | 3.04 | 1.86 | 1.68 |
| *Patescibacteria* | 1.64 | 1.96 | 2.88 | 4.28 | 0.99 |
| *Bacteroidetes* | 0.8 | 1.32 | 1.64 | 1.54 | 1.91 |
| *Verrucomicrobia* | 1.3 | 1.15 | 1.06 | 0.95 | 2.11 |
| *Firmicutes* | 0.23 | 0.31 | 0.32 | 0.47 | 1.11 |

**Supplementary Table 2** Relative abundance of fungal community in PG treated soil at the phylum level.

| Phyla | Control | P1 | P2 | P3 | P4 |
| --- | --- | --- | --- | --- | --- |
| *Ascomycota* | 41.53 | 39.64 | 37.66 | 40.05 | 30.54 |
| *Mortierellomycota* | 28.46 | 25.05 | 30.06 | 25.51 | 48.36 |
| *Basidiomycota* | 23.21 | 27.08 | 23.35 | 26.37 | 17.32 |
| *Chytridiomycota* | 0.13 | 0.34 | 0.09 | 0.14 | 0.01 |
| *Glomeromycota* | 0.14 | 0.04 | 0.15 | 0.03 | 0.01 |
| *Rozellomycota* | 0.03 | 0.16 | 0.01 | 0.06 | 0.03 |
| *Mucoromycota* | 0.03 | 0.03 | 0.02 | 0.04 | 0.10 |
| *Entorrhizomycota* | 0.02 | 0.02 | 0.02 | 0.01 | 0.01 |
| *Basidiobolomycota* | 0.00 | 0.00 | 0.06 | 0.00 | 0.00 |
| *Kickxellomycota* | 0.00 | 0.01 | 0.00 | 0.00 | 0.01 |

**Supplementary Table 3** Correlation between environmental factors and bacterial community structure

|  | RDA1 | RDA2 | r2 | Pr(>r) |
| --- | --- | --- | --- | --- |
| pH(H_2_O) | 0.348 | -0.937 | 0.273 | 0.164 |
| pH(KCl) | 0.987 | 0.160 | 0.904 | 0.001 |
| EC | 0.957 | 0.290 | 0.953 | 0.001 |
| NH_4_^+^-N | 0.998 | -0.065 | 0.839 | 0.003 |
| NO_3_^-^-N | 0.359 | 0.933 | 0.561 | 0.006 |
| Ca^2+^ | 0.903 | 0.429 | 0.931 | 0.001 |
| F^-^ | 1.000 | 0.026 | 0.881 | 0.001 |

**Supplementary Table 4** Correlation between environmental factors and fungal community structure

|  | RDA1 | RDA2 | r2 | Pr(>r) |
| --- | --- | --- | --- | --- |
| pH (H_2_O) | 0.993 | 0.117 | 0.145 | 0.400 |
| pH (KCl) | 0.999 | 0.053 | 0.785 | 0.001 |
| EC | 1.000 | -0.018 | 0.700 | 0.003 |
| NH_4_^+^-N | 0.988 | 0.153 | 0.809 | 0.002 |
| NO_3_^-^-N | 0.925 | -0.379 | 0.018 | 0.891 |
| Ca^2+^ | 0.992 | -0.127 | 0.5728 | 0.008 |
| F^-^ | 0.996 | 0.094 | 0.829 | 0.001 |

**Supplementary Table 5** Heavy metal content in PG and soil (mg/Kg)

| Heavy metal elements | PG | Control | GB8172-87 | GB4284-84 |
| --- | --- | --- | --- | --- |
| Cd | 0.023 | 0.125 | ≤ 5 | ≤ 3 |
| Hg | 0.277 | 0.087 | ≤ 5 | ≤ 5 |
| As | 12.26 | 15.33 | ≤ 75 | ≤ 30 |
| Pb | 7.127 | 16.31 | ≤ 300 | ≤ 100 |
| Cr | 5.527 | 58.43 | ≤ 600 | ≤ 300 |

**Supplementary Table 6** Chemical composition of PG

| sample | CaO | SO_3_ | Total P_2_O_5_ | Water soluble P_2_O_5_ | Eutectic P_2_O_5_ | Total F | Water soluble F | Al_2_O_3_ | Total organic carbon (TOC) | Loss on ignition |
| --- | --- | --- | --- | --- | --- | --- | --- | --- | --- | --- |
|  | % | | | | | | | | | |
| PG | 30.54 | 42.22 | 1.85 | 0.81 | 0.62 | 0.84 | 0.08 | 0.21 | 0.40 | 21.81 |
